# Cerebral hemodynamics during neonatal transition according to mode of delivery

**DOI:** 10.1038/s41598-021-98932-7

**Published:** 2021-09-29

**Authors:** Aya Morimoto, Shinji Nakamura, Masashiro Sugino, Kosuke Koyano, Noriko Fuke, Makoto Arioka, Yasuhiro Nakao, Ami Mizuo, Mari Matsubara, Yuta Noguchi, Katsufumi Nishioka, Takayuki Yokota, Ikuko Kato, Yukihiko Konishi, Sonoko Kondo, Jun Kunikata, Takashi Iwase, Saneyuki Yasuda, Takashi Kusaka

**Affiliations:** 1 Department of Pediatrics, Faculty of Medicine, Kagawa University, 1750-1 Miki-cho, Kita-gun, Kagawa 761-0793 Japan; 2 Division of Neonatology, Shikoku Medical Center for Children and Adults, Zentsuji, Japan; 3 Maternal Perinatal Center, Faculty of Medicine, Kagawa University, Kita-gun, Japan; 4 Post Graduate Clinical Education Center, Kagawa University Hospital, Kita-gun, Japan; 5 Clinical Research Support Center, Kagawa University Hospital, Kita-gun, Japan

**Keywords:** Applied optics, Paediatric research

## Abstract

Cerebral haemodynamics during the immediate transition period in neonates may differ depending on whether delivery is vaginal or by caesarean section. However, these differences have never been confirmed by near-infrared time-resolved spectroscopy (TRS). Therefore, the purpose of this study was to compare cerebral blood volume (CBV) and cerebral haemoglobin oxygen saturation (ScO_2_) between healthy term neonates by mode of delivery. Subjects were 31 healthy term neonates who did not require resuscitation. Thirteen neonates were delivered vaginally (VD group) and 18 were delivered by elective caesarean section (CS group). Absolute oxyhaemoglobin, deoxyhaemoglobin, and total haemoglobin concentrations were measured continuously by TRS; oxyHb × 100/totalHb (ScO_2_) (%) and CBV (mL/100 g brain tissue) were also calculated. Measurements were started as soon as possible after birth, obtained from 1 to 2 min after birth, and continued until 15 min after birth. CBV was significantly higher in the VD group than in the CS group in the 4 min after birth but not thereafter. There were no significant between-group differences in ScO_2_ and SpO_2_. These findings indicate that there is a difference in cerebral haemodynamic patterns in the first 4 min after delivery between term neonates by mode of delivery when CBV is monitored by TRS.

## Introduction

Newborn infants are subjected to a dramatic environmental change at the time of birth and must adapt rapidly to life in the extrauterine environment. The transition from foetal to newborn life is a major physiological challenge that all humans must overcome to survive. In almost all cases, the transition to pulmonary breathing at birth occurs without impediment.

However, adaptation of cardiac function after birth is affected by many factors during the perinatal period. Differences in foetal perfusion related to the mode of delivery (vaginal or caesarean section) may influence foetal haemodynamics. Umbilical arterial adenosine and catecholamine concentrations have been found to differ significantly between neonates born vaginally and those born by caesarean section^1^, suggesting that early neonatal cardiovascular patterns may differ according to mode of delivery.

The neonatal resuscitation guideline now recommends routine monitoring with pulse oximetry/electrocardiography^2^. These two non-invasive continuous monitoring methods enable measurement of the arterial oxygen saturation (SpO_2_) and heart rate. However, few studies have investigated whether there are any differences in neonatal haemodynamic patterns according to mode of delivery when using these parameters^3–5^.

There is increasing interest in additional monitoring of neonates during the immediate transition period using near-infrared spectroscopy (NIRS). NIRS is non-invasive and can detect changes in oxyhaemoglobin (oxyHb) and deoxyhaemoglobin (deoxyHb) concentrations in cerebral tissue. There have been several reports on use of NIRS to measure cerebral oxygenation via the tissue oxygenation index or regional saturation of oxygen in neonates during the immediate transition after birth^6–12^. We have previously reported a difference in the pattern of cerebral haemoglobin (Hb) oxygen saturation (ScO_2_) during the first 15 min after birth according to whether delivery is vaginal or by caesarean section^13,14^. In contrast, Urlesberger et al. found no differences in regional oxygen saturation in the brain according to mode of delivery^6^.

NIRS can also measure the changes in cerebral Hb concentration (tHb) and cerebral blood volume (CBV), with some work showing a decrease in CBV in term neonates in the first 15 min after birth^7^. Near-infrared time-resolved spectroscopy (TRS) is a unique method for calculating not only quantitative ScO_2_ but also CBV; it uses a light-absorption coefficient without inducing changes in light-absorbing materials, such as oxygenated Hb and indocyanine green, because the respective light-reduced scattering and absorption coefficients can be determined by resolving the photon diffusion equation^15^. We have already demonstrated that TRS can stably measure cerebral haemodynamics despite the dramatic physiological changes that occur during labour in the transition period^16^. However, there are still no reports on the differences in absolute CBV patterns in healthy term neonates according to mode of delivery.

In this study, we hypothesised that changes in CBV would be less in infants delivered by elective CS than in those who delivered vaginally and, investigated the changes in absolute CBV during the immediate transition period in healthy infants according to whether delivery was vaginal or by elective caesarean section and how the CBV pattern differed between the two modes of delivery.

## Results

Thirty-seven healthy term infants were delivered vaginally (n = 17) or by elective caesarean section (n = 20) at Kagawa University Hospital during the study period. Two neonates delivered by caesarean section were excluded because of the need for respiratory support after delivery and four neonates delivered vaginally were excluded because of an abnormal μs′ value, leaving 31 infants for analysis (VD group, n = 13; CS group, n = 18) (Table 1). The gestational age at delivery was 37–41 weeks and the Apgar scores at 1 min were ≥ 7.

**Table 1:** Demographic and clinical data of term neonates according to mode of delivery.

	Vaginal delivery (n = 13)	Caesarean section (n = 18)	*P* value
Gestational age (weeks)	39.7 (1.0)	38.5 (1.0)	0.002
Birth weight (g)	3281 (383)	2806 (378)	0.002
Apgar score at 1 min	8 (0)	8 (0.4)	0.64
Apgar score at 5 min	9 (0.3)	9 (0.3)	0.76
pH in umbilical artery	7.314 (0.04)	7.285 (0.05)	0.12
Venous haemoglobin at 2 h (g/dL)	18.5 (2.4)	18.1 (2.0)	0.59

Data are show as the mean (standard deviation).

Neonates delivered by elective caesarean section were significantly lighter than those delivered vaginally because elective caesarean section is performed from a gestational age of 38 weeks in our hospital.

**Figure 1:**
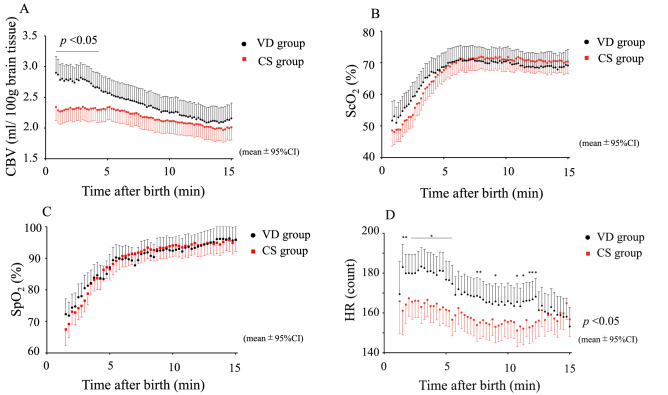
Time course of CBV (**A**), ScO_2_ (**B**), SpO_2_ (**C**), and HR (**D**) during the first 15 min after birth in healthy neonates in the VD group (black) and the elective CS group (red). The values are shown as the mean (95% confidence interval [CI] of the mean); *P* < 0.05, group comparisons by linear mixed model. *CBV* cerebral blood volume, *CS* caesarean section, *ScO*_*2*_ cerebral haemoglobin oxygen saturation, *SpO*_*2*_ arterial oxygen saturation, *VD* vaginal delivery.

CBV peaked at about 1–3 min after birth, gradually decreased until 15 min, and then stabilised in both groups. CBV was significantly higher in the 4 min after birth in the VD group than in the CS group (difference = VD − CS; mean [95%CI]: 0.3 [0.0–0.7], P = 0.039; linear mixed model) [(Fig. 1A) VD/CS group mean [95% CI] mL/100 g brain tissue: 1.5 min, 2.8 [2.5–3.0]/2.3 [2.1–2.5]; 2 min, 2.8 [2.6–3.0]/2.3 [2.1–2.5]; 3 min, 2.8 [2.6–3.0]/2.4 [2.1–2.6]; 4 min, 2.7 [2.4–2.9]/2.3 [2.1–2.5]; 5 min, 2.6 [2.3–2.8]/2.3 [2.1–2.5]; 10 min, 2.3 [2.0–2.5]/2.1 [1.9–2.3]; and 15 min, 2.2 [1.9–2.4]/2.0 [1.8–2.2]].

ScO_2_ shows the same pattern as SpO_2_, namely, a gradual increase, a peak at 5–10 min, and then stabilizing thereafter [(Fig. 1B) VD/CS mean [95%CI]%: 1.5 min, 53.3 [48.6–58.0]/48.8 [45.0–52.6]; 2 min, 56.2 [51.8–60.7]/51.9 [48.1–55.7]; 3 min, 61.8 [57.4–66.3]/57.2 [53.5–61.0]; 4 min, 67.2 [62.7–71.6]/64.0 [60.2–67.8]; 5 min, 68.7 [64.2–73.1]/67.7 [64.0–71.5]; 10 min, 70.4 [66.0–74.9]/71.0 [67.2–74.7]; and 15 min, 69.1 [64.0–74.2]/70.4 [66.5–74.3]] There was no significant difference in ScO_2_ between the two groups (p-values by linear mixed model: 1.5 min, 0.14; 2 min, 0.14; 3 min, 0.12; 5 min, 0.75; 10 min, 0.85; and 15 min, 0.70). There was also no significant difference between the groups in SpO_2_ (Fig. 1C).

HR peaked at about 1–3 min after birth, gradually decreased until about 15 min in the VD group and 8 min in the CS group, and then stabilised in both groups. HR was significantly higher until 5 min after birth in the VD group compared with the CS group (difference = VD − CS; mean [95%CI]: 15.1 [2.8–27.5], P = 0.017; linear mixed model), and then tended to be higher in the VD group until about 12 min after birth [(Fig. 1D) VD/CS group mean [95% CI] mL/100 g brain tissue: 1.5 min, 183.0 [171.4–194.6]/161.1 [149.0–173.2]; 2 min, 180.0 [170.5–189.5]/167.4 [158.4–176.4]; 3 min, 183.3 [173.8–192.8] / 162.9 [154.7–171.2]; 4 min, 180.2 [170.9–189.5]/163.4 [155.1–171.6]; 5 min, 176.7 [167.4–186.0]/161.6 [153.5–169.7]; 10 min, 165.9 [156.8–175.0]/155.4 [147.3–163.5]; 12 min, 166.8 [157.7–175.9] / 153.5 [145.3–161.6]; and 15 min, 153.2 [143.7–162.7]/156.8 [148.2–165.4]].

Next, we examined the concentrations of oxyHb and deoxyHb at 30-s intervals from 2 to 5 min after birth by two-way analysis of variance (with repeated measures) between the VD and CS groups. The oxyHb concentration tended to be higher in the VD group than in the CS group at 2.5–3.5 min after birth. However, there was no difference in deoxyHb concentration between the groups.

## Discussion

The two main findings of this study were as follows: (1) CBV was significantly higher in the VD group than in the CS group in the first 4 min after delivery but decreased thereafter during the first 15 min in both groups and (2) there was an increase in ScO_2_ including SpO_2_, but it was not significantly different between these delivery modes. This is the first study to show that CBV is a cerebral haemodynamic parameter that is significantly affected by mode of delivery in the neonate. Furthermore, this was the first time TRS using probes with a bent tip was applied to neonates and the procedure was determined to be feasible during the transition period after birth.

Schwaberger et al. reported a decrease in CBV within 15 min of birth in healthy neonates delivered by caesarean section, and Noori et al. demonstrated a decrease in cerebral blood flow (CBF) in preterm infants after birth using Doppler sonography^17^. Our study showed a decrease in CBV within 15 min after birth not only in CS, but also in VD, consistent with those previous studies. These decreases in CBF and CBV during postnatal transition may have significant clinical implications, although the relation between CBF and CBV in neonates remains unclear. Schwaberger et al. speculated that the cause of this reduction in CBF was an increase in arterial oxygen content and/or changes in shunting through the ductus arteriosus during the transition period^7^. We speculate that another factor that may contribute to the decrease in CBV within the first 15 min after birth is clamping of the umbilical cord postpartum. In a study in newborn lambs by Polglase et al., CBF increased rapidly within the first 60 s after cord clamping and remained elevated throughout the entire ventilation period. The authors attributed the rapid increase in CBF to a combination of haemodynamic responses to clamping of the umbilical cord (i.e., removal of the capacitance placental circulation)^18^.

In this study, CBV was significantly higher in the VD group than in the CS group during the first 4 min after birth but was similar in the two groups by 10–15 min after birth. A possible explanation for the higher CBV within the first 4 min after birth could be the hypoxia that occurs during labour and passage through the birth canal, which causes the marked antepartum difference between normal vaginal delivery and elective caesarean section. There are a few reports on cerebral haemodynamics during labour^19–21^. Uterine contractions result in a decline in foetal PaO_2_ by approximately 25%, but the majority of appropriately grown, healthy, term foetuses are able to withstand this effect. This hypoxia stimulated the brain stem to increase both the parasympathetic and sympathetic outflow, which induces profound peripheral vasoconstriction. This leads to hypertension and increases the descending aortic pressure, which in turn increases the right ventricular afterload, encouraging passage of blood from the right atrium through the foramen ovale into the left atrium and then the left ventricle, thereby increasing blood flow into the ascending aorta as well as cerebral blood flow^22^. In one previous report, CBV was increased by labour and pushing during the second stage of labour, and the change in CBV after birth in our study is consistent with that report^19^. Such hypoxia to the degree caused by labour is not a serious problem in the process of birth, because the foetus responds to physiological compensations; therefore, blood gas data after birth was in the normal range. This is similar to previous reports that UApH does not differ by mode of delivery^1,6^. However, elective CS without labour might not show these compensatory reactions as is the case with labour before birth in vaginal delivery. CBF might not increase and thus there will be less of an increase in CBV.

TRS can determine the absolute total Hb value, which consists of oxyHb and deoxyHb. In this study, the finding of a higher oxyHb in our VD group indicates that vasodilation in the cerebral arterial circulation might be more marked in neonates delivered vaginally than in those delivered by elective caesarean section. Furthermore, sympathetic activation by certain stressors, such as mechanical compression of the head, can promote incretion of catecholamines, and the foetus becomes ready for birth while maintaining its cerebral haemodynamics. There are some reports on the foetal response to hypoxia that focus on cerebral haemodynamics and oxygenation^23–25^. One study showed that when acute hypoxia is detected by chemoreceptors in the carotid body, there is an increase in sympathetic and parasympathetic outflow from the brain stem, which triggers an increase in CBF and cerebral perfusion^22^.

Although there was no significant difference in ScO_2_, between the two delivery modes, it tended to be higher in the VD group than in the CS group at 5 min after birth. Pichler et al. found significantly higher ScO_2_ in a VD group than in a CS group at 4 and 5 min after birth. They speculated that this difference in ScO_2_ may reflect differences in CBF, likely as a result of cerebral autoregulation, which may be caused by the difference in arterial oxygen content between VD and CS^8^. The reason why there was no significant difference in ScO_2_ in our study within 5 min after birth was that oxyHb tended to be higher in VD than in CS, but deoxyHb had the same decreasing pattern in both groups.

HR was higher in the VD group than in the CS group in this study, which is consistent with previous reports^8,26^. In contrast, Almaazmi et al. reported that HR was almost identical in healthy term infant regardless of mode of delivery^9^. We speculate that the lack of significant differences between the two groups may be attributable to arrested labour in their study. CBV depends on cardiac output and vascular resistance. When HR is used as a measure of cardiac output, the difference in HR suggests that CBV differs by mode of delivery. However, in this study, we measured only cerebral haemodynamics and oxygenation; we did not measure systemic haemodynamics except for HR. We checked the venous blood gas in all neonates 2 h after birth and calculated CBV but could not obtain neonatal blood gas measurements immediately after birth as an indicator of the stress caused by delivery. Furthermore, the number of neonates was too small to define a range of CBV. The early CBV data obtained at around 2 min did not provide enough information about the initial process of cerebral haemodynamic adaptation to the extrauterine environment. In our next study, we will measure these data earlier such as before cord clamping throughout delivery. Healthy neonates born by vaginal delivery had a significantly higher CBV than those born by elective caesarean section in the immediate transition period, although there was no significant difference in ScO_2_. This difference in CBV between the two delivery modes may reflect a difference in cerebral arterial vasodilation, which may be more pronounced in response to hypoxia during passage through the birth canal in neonates born by vaginal delivery. CBV has the potential to be a useful parameter for understanding the initial process of cerebral haemodynamic adaptation after birth.

## Methods

This prospective observational study was performed at Kagawa University Hospital and involved term neonates (gestational age > 37 weeks, birth weight > 2300 g) born by vaginal delivery or elective caesarean section, which was performed under spinal anaesthesia, between October 2012 and April 2019.

**Figure 2:**
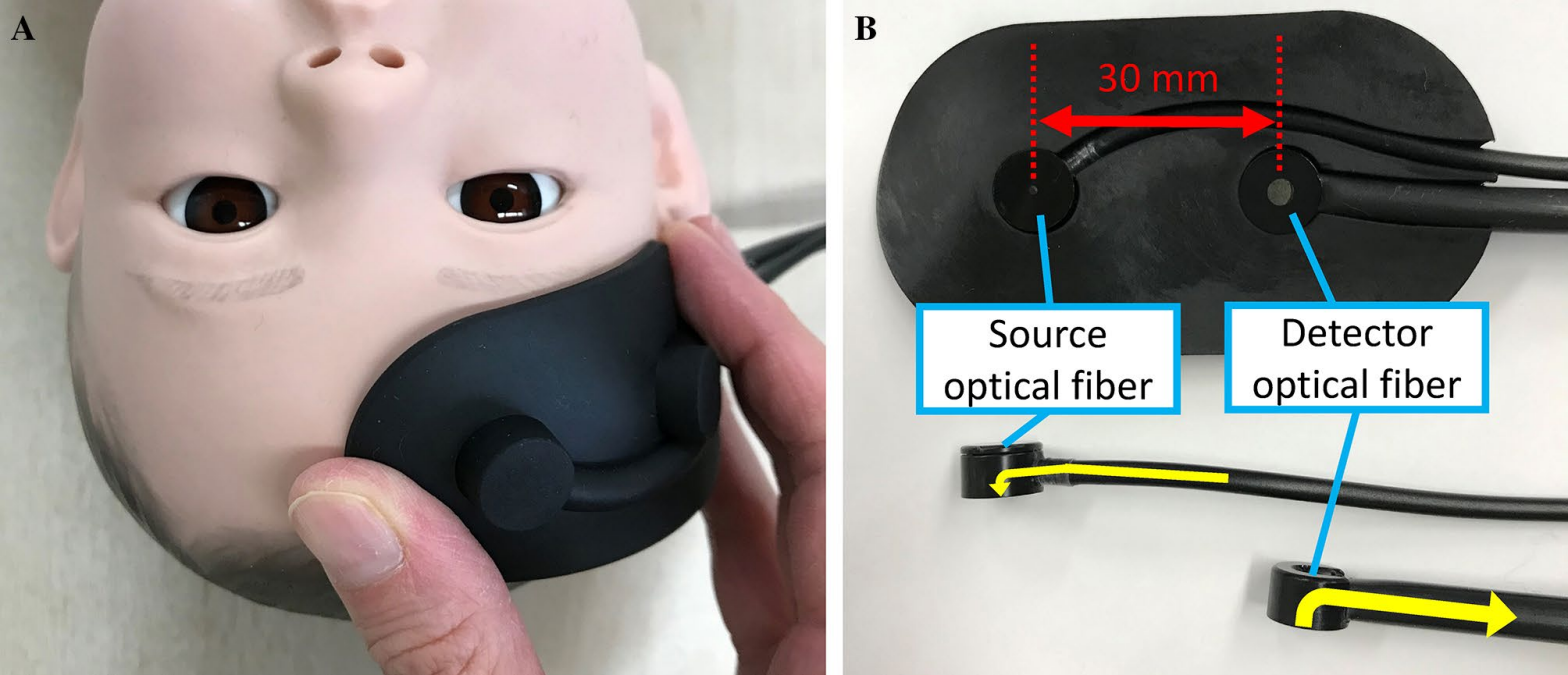
Time-resolved spectroscopy attachment diagram on a simulated infant (mannequin). (**A**) The light source and detection optodes were positioned on the frontal region. (**B**) The interoptode distance was 30 mm. The yellow arrow indicates the optical direction of the 90° bent tip.

After cord clamping, which is performed routinely after 30 s, the neonates were placed in a supine position and breathed room air on the resuscitation table under an overhead heater. A neonatologist observed the transition of each newborn infant and recorded Apgar scores at 1 and 5 min. Resuscitation was performed in accordance with the Neonatal Cardiac Pulmonary Resuscitation 2015 guidelines. A portable three-wavelength TRS system (TRS-21; Hamamatsu Photonics K.K., Hamamatsu, Japan) was used. As soon as possible after delivery, another neonatologist attached the TRS optical probe to the newborn’s forehead on the right side and obtained measurements continuously for 15 min (Fig. 2A). At the same time, a transcutaneous pulse oximeter (Nellcor; Covidien, Mansfield, MA) was applied to the right hand for simultaneous measurement of SpO_2_.

The following exclusion criteria were applied: (1) a need for any form of respiratory support, such as oxygen, continuous positive airway pressure, or artificial ventilation; (2) hospitalisation due to hypoglycaemia or infection; (3) emergency caesarean section because of obstructed labour and foetal distress; and (4) abnormal optical properties in reference to our previous published data^27^, namely, a light-reduced scattering value > 9.0/cm (2SD).

### Near-infrared time-resolved spectroscopy

TRS uses a time-correlated single-photon counting technique for detection. The system was controlled by a computer through a digital I/O interface that consisted of a three-wavelength (762, 800, and 836 nm) picosecond light pulser as the pulse light source, a photon-counting head for single-photon detection, and signal-processing circuits for time-resolved measurement. The re-emission profiles observed at each measurement point were fitted with the photon diffusion equation proposed by Patterson et al.^28^ to calculate the absorption coefficient (μa) and the reduced scattering coefficient (μs′) values of the head at wavelengths of 762, 800, and 836 nm.

In each iterative calculation, the photon diffusion equation was calculated in reflectance mode and was convoluted with the instrumental response; it was then fitted to the observed re-emission profile. After determination of the µa and µs′ values at the three wavelengths, the oxyHb and deoxyHb concentrations were calculated from their respective absorption coefficients using the following equations, based on the assumption that the background absorption was due to 85% (by volume) water.

$$\begin{aligned}\upmu {\mathrm{a}}_{762nm} &= {\varepsilon }_{762nm}^{oxyHb}\left[oxyHb\right]+ {\varepsilon }_{762nm}^{deoxyHb}\left[deoxyHb\right]+\upmu{\mathrm{a}}_{762nm}^{background}\\ \upmu {\mathrm{a}}_{800nm}&= {\varepsilon }_{800nm}^{oxyHb}\left[oxyHb\right]+ {\varepsilon }_{800nm}^{deoxyHb}\left[deoxyHb\right]+\upmu{\mathrm{a}}_{800nm}^{background}\\ \upmu{\mathrm{a}}_{836nm}& = {\varepsilon }_{836nm}^{oxyHb}\left[oxyHb\right]+ {\varepsilon }_{836nm}^{deoxyHb}\left[deoxyHb\right]+\upmu{\mathrm{a}}_{836nm}^{backgroungd} \end{aligned}$$

In these equations, $\varepsilon_{{\uplambda {\text{nm}}}}^{x}$ is the extinction coefficient at λ nm, and [oxyHb] and [deoxyHb] are the concentrations of oxyHb and deoxyHb, respectively.

We used a source optical fibre bundle with a diameter of 1 mm and a detector optical fibre bundle with a diameter of 3 mm, both with a 90° bent tip and numerical aperture of 0.29 (Fig. 2B). The light emission and detection optodes were positioned on the frontal region at an interoptode distance of 30 mm. The total cerebral Hb (totalHb) concentration, ScO_2_, and CBV values were calculated as follows:

$$\begin{aligned} & \left[ {{\text{totalHb}}} \right] = \left[ {{\text{oxyHb}}} \right] + \left[ {{\text{deoxyHb}}} \right], \\ & {\text{ScO}}_{{2}} \left( \% \right) = (\left[ {{\text{oxyHb}}} \right]/\left( {\left[ {{\text{oxyHb}}} \right] + \left[ {{\text{deoxyHb}}} \right]} \right) \times {1}00, \\ & {\text{CBV}}\;\left( {{\text{mL}}/{1}00{\text{ g brain tissue}}} \right) = \left[ {{\text{totalHb}}} \right] \times {\text{MW}}_{{{\text{Hb}}}} \times { 1}0^{{ - {6}}} /\left( {{\text{tHb}} \times {1}0^{{ - {2}}} \times {\text{Dt}} \times {1}0} \right), \\ \end{aligned}$$

where [ ] indicates the Hb concentration (µM), MW_Hb_ is the molecular weight of Hb (64,500), tHb is the venous Hb concentration (g/dL), and Dt is the brain tissue density (1.05 g/mL).

All neonates underwent blood gas analysis, and CBV was calculated from the venous Hb concentration at 2 h after birth.

### Statistical analysis

Between-group differences in patient characteristics were analysed using the *t*-test. The mean CBV and ScO_2_ values were calculated at 10-s intervals and the SpO_2_ data were measured at 15-s intervals for 15 min after birth.

For the parameters measured over time (CBV, ScO_2_, SpO_2_, and HR), a linear mixed model was used to analyse the difference between the CS and VD groups at each time point. This is because there are differences in the number of time points measured in different cases. A model was constructed with the parameters measured over time as the dependent variable, subjects as a random factor, and time, groups, and their interaction terms (time × group) as fixed factors. For each estimate, the least squares mean and its 95% confidence interval (CI) were calculated. Statistical analyses of CBV, ScO_2,_ SpO_2_, and HR were performed using SPSS for Windows version 24.0 (IBM Japan, Tokyo, Japan). A two-sided *P* value < 0.05 was considered statistically significant.

We then compared oxyHb and deoxyHb values obtained within the first 5 min by repeated measures two-way analysis of variance followed by post hoc analysis with Sidak’s multiple comparisons test to determine the time points when differences were evident. Statistical analyses of oxyHb and deoxyHb were performed using GraphPad Prism 8 (GraphPad Software, La Jolla, CA). A *P* value < 0.05 was considered statistically significant.

### Ethical approval and informed consent

The study was approved by the Regional Committee on Biomedical Research Ethics of Kagawa University (approval number: H29-042) and conducted in accordance with the Declaration of Helsinki. The parents of all neonates enrolled in the study provided written informed consent before delivery after receiving a full explanation of the research.

## Data Availability

The datasets generated during and/or analysed during the current study are available from the corresponding author on reasonable request.
